# Differential regulation of HIF-mediated pathways increases mitochondrial metabolism and ATP production in hypoxic osteoclasts

**DOI:** 10.1002/path.4159

**Published:** 2013-03-13

**Authors:** Karl J Morten, Luned Badder, Helen J Knowles

**Affiliations:** 1 Nuffield Department of Obstetrics and Gynaecology, The Womens Centre, John Radcliffe HospitalOxford, UK; 2 Botnar Research Centre, Nuffield Department of Orthopaedics, Rheumatology and Musculoskeletal Sciences, University of Oxford, Nuffield Orthopaedic CentreOxford, UK

**Keywords:** hypoxia, osteoclast, hypoxia-inducible factor, ATP, metabolism

## Abstract

Inappropriate osteoclast activity instigates pathological bone loss in rheumatoid arthritis. We have investigated how osteoclasts generate sufficient ATP for the energy-intensive process of bone resorption in the hypoxic microenvironment associated with this rheumatic condition. We show that in human osteoclasts differentiated from CD14^+^ monocytes, hypoxia (24 h, 2% O_2_): (a) increases ATP production and mitochondrial electron transport chain activity (Alamar blue, O_2_ consumption); (b) increases glycolytic flux (glucose consumption, lactate production); and (c) increases glutamine consumption. We demonstrate that glucose, rather than glutamine, is necessary for the hypoxic increase in ATP production and also for cell survival in hypoxia. Using siRNA targeting specific isoforms of the hypoxia-inducible transcription factor HIF (HIF-1*α*, HIF-2*α*), we show that employment of selected components of the HIF-1*α*-mediated metabolic switch to anaerobic respiration enables osteoclasts to rapidly increase ATP production in hypoxia, while at the same time compromising long-term survival. We propose this atypical HIF-driven metabolic pathway to be an adaptive mechanism to permit rapid bone resorption in the short term while ensuring curtailment of the process in the absence of re-oxygenation. Copyright © 2013 Pathological Society of Great Britain and Ireland. Published by John Wiley & Sons, Ltd.

## Introduction

Osteoclasts are large, non-proliferative, multinucleated cells responsible for the resorptive component of bone remodelling. Osteoclast-mediated osteolysis is a diagnostic feature of rheumatoid arthritis (RA), contributing to progressive disability and predictive of poor prognosis 1. Osteoclasts bind bone via *α*v*β*3 integrin, then form an F-actin-rich seal to isolate a resorptive compartment above the osseous substrate. Resorption is initiated by active transport of protons across the bone-apposing membrane by vacuolar H^+^ ATPase, a process also supported by Na,K-ATPase, Ca-ATPase and gastric H,K-ATPase 2. This acidifies the resorptive compartment, exposing the organic matrix to proteolytic enzymes also secreted from the osteoclast. Osteoclasts are additionally highly motile and these processes render bone resorption an energy-intensive process with a high demand for ATP 3,4.

Hypoxia is another feature of the rheumatoid joint and also a poor prognostic indicator 5. Cellular adaptation to hypoxia generally entails switching to anaerobic metabolism; reducing ATP production to prevent accumulation of reactive oxygen species (ROS) 6. This survival mechanism is largely mediated by the hypoxia-inducible factor (HIF-1, HIF-2) family of transcription factors. Post-translational stabilization of the *α*-subunit of HIF-1 and HIF-2 under hypoxia transactivates numerous downstream genes, including those mediating the switch to initiate metabolic adaptation to hypoxia and maintain energy/redox homeostasis 7.

First, HIF triggers a cytochrome *c* oxidase subunit switch (COX4-1 to COX4-2), which increases the efficiency of complex IV of the mitochondrial electron transport chain (ETC) with respect to the amounts of ATP and ROS produced 8. Once this is insufficient to maintain homeostasis, HIF stimulates glucose transporter and glycolytic enzyme expression to increase glycolytic flux 9. Third, HIF inhibits pyruvate dehydrogenase (PDH), the mitochondrial enzyme that converts pyruvate into acetyl CoA, by increasing expression of PDH kinase (PDK) which phosphorylates and inactivates PDH 10,11. This reduces flux through the mitochondrial tricarboxylic acid (TCA) cycle and ETC and reduces accumulation of ROS. Fourth, HIF induces expression of BCL2/adenovirus E1B 19 kDa interacting protein 3 (BNIP3), which competes with Beclin-1 for binding to Bcl-2, releasing Beclin-1 to stimulate mitochondrial autophagy and also reduce accumulation of ROS 12.

Hypoxia exerts various effects on osteoclasts. It reduces the viability of mature osteoclasts 13,14, increases osteoclast differentiation when combined with periods of re-oxygenation 13–16 and increases bone resorption in a HIF-1*α*-dependent manner 13–17. Osteoclasts exhibit elevated expression of TCA cycle and oxidative phosphorylation enzymes 18, high rates of oxygen consumption 19 and numerous mitochondria 20, suggesting that high mitochondrial metabolic activity drives ATP production in these cells. The preferred substrate is glucose (via glycolysis) 19,21,22, although fatty acid oxidation has been implicated 23. Given the high energy requirement for resorption, it could be presumed that increased resorption under hypoxia would increase cellular demand for ATP. However, switching to anaerobic glycolysis would not be expected to support this increased demand.

We have investigated the metabolic requirements of hypoxic osteoclasts to determine how they generate the requisite ATP for increased bone resorption. Identification of key components of this pathway might highlight possible therapeutic targets for amelioration of pathological bone resorption conditions.

## Methods

### Reagents

Tissue culture reagents were from Lonza (Wokingham, UK), except FBS (Invitrogen, Paisley, UK), M-CSF (R&D Systems, Abingdon, UK) and RANKL (PeproTech, London, UK). Compound C was from Merck (Feltham, UK). Unless stated otherwise, other reagents were from Sigma (Poole, UK). This study was approved by the Oxford Clinical Research Ethics Committee (C01.071) and the Oxford Musculoskeletal BioBank.

### Cell culture

Cells were cultured in *α*-MEM (without ribonucleosides/deoxyribonucleosides), 10% FBS, l-glutamine (2 mm), penicillin (50 IU/ml) and streptomycin sulphate (50 µg/ml). Peripheral blood mononuclear cells were isolated from buffy coat using Histopaque. Positively selected CD14^+^ monocytes (AutoMACS cell separator; Miltenyi Biotec, Bisley, UK) were seeded onto tissue culture plates, glass slides or dentine slices at 10^6^ cells/well of a 24-well plate. Non-adherent cells were removed and cultures supplemented with (a) M-CSF (25 ng/ml) for monocyte culture (≤ 72 h) or (b) M-CSF (25 ng/ml) + RANKL (50 ng/ml) every 3–4 days for osteoclast culture, with experiments performed on days 13–17. Primary human osteoblasts were obtained by outgrowth from cancellous bone chips removed during surgery. Hypoxic exposure (2% O_2_, 5% CO_2_, balance N_2_) was performed in a MiniGalaxy incubator (RS Biotech, Irvine, UK). Vitronectin receptor (VNR) was detected using a CD51/61 monoclonal antibody (AbD Serotec, Oxford, UK). Multinucleated cells containing ≥ 3 nuclei were considered osteoclasts. Resorption pits were stained (0.5% toluidine blue), photographed and quantified using ImageJ.

### Mitochondrial function

Intracellular ATP was assayed using CellTiter Glo (Promega, Southampton, UK). Mitochondrial dehydrogenase activity within the ETC was assessed using Alamar blue (AbD Serotec) and normalized to cell number using crystal violet, which stains nuclei independently of cellular metabolic status. Oxygen consumption was assessed using MitoXpress-Xtra-HS (Luxcel Biosciences, Cork, Ireland), a porphyrin-based phosphorescent oxygen-sensitive probe. 16 h prior to assay, osteoclasts were transferred into fresh culture medium or medium lacking glucose but supplemented with 1 mm pyruvate. Probe (10 µl) was added and the cells equilibrated at 20% or 2% O_2_. The assay was read using a FLUOstar Omega plate reader with ACU (BMG Labtech, Aylesbury, UK), held at 20% or 2% O_2_, the maximal rate of oxygen consumption being proportional to the change in probe fluorescence during the linear phase of the assay 24 (see Supplementary material, additional methodology for O_2_ consumption assay). To assess relative mitochondrial number, nonyl-acridine orange was applied to cells in 5% FBS/PBS (50 nm, 30 min, 37°C), then visualized by fluorescence microscopy. Staining intensity was quantified in ImageJ and normalized to number of nuclei and osteoclast area.

### PCR

RNA was extracted in TRI reagent, DNase-treated and reverse-transcribed (SuperScript VILO cDNA Synthesis Kit, Invitrogen). Real-time PCR was performed with Express SYBR GreenER qPCR Supermix Universal (Invitrogen) and QuantiTect primers (Qiagen, Crawley, UK). Expression was normalized to *ACTB*.

### Western blot

Cells were homogenized in lysis buffer [6.2 m urea, 10% glycerol, 5 mm dithiothreitol (DTT), 1% sodium dodecyl sulphate (SDS), protease inhibitors] or phospho-lysis buffer [1 mm EDTA, 1 mm phenylmethylsulphonyl fluoride (PMSF), 1 mm Na_3_VO_4_, 1 mm NaF in PBS]. Primary antibodies were against SOD2 (ab13533), porin (clone 31HL; Abcam, Cambridge, UK), BNIP3 (clone Ana40), COX IV isoform 1, COX IV isoform 2 (Novus Biologicals, Cambridge, UK), HIF-1*α* (clone 54; BD Biosciences), AMPK*α* (23A3), phospho-AMPK*α* (Thr172, 40H9; Cell Signalling Technology, Danvers, MA, USA) and *β*-tubulin (TUB2.1).

### Metabolites

Glucose and glutamine were measured using the Glucose (GO) Assay Kit and Glutamine/Glutamate Determination Kit. Lactate was assayed in heat-inactivated medium by the increase in absorbance (340 nm) as NAD^+^ was converted to NADH in the presence of 0.32 m glycine, 0.32 m hydrazine, 9.6 mm NAD^+^ and 3 U/ml lactate dehydrogenase. The results were normalized to osteoclast number. Intracellular neutral lipid was detected in formalin-fixed cells washed with 60% isopropanol, air-dried, incubated in Oil Red O (70 mm) and washed and photographed in distilled water.

### PDH activity

The PDH Enzyme Activity Microplate Assay (Abcam) was used to assess the ability of immunocaptured PDH to convert pyruvate to acetyl-CoA by following the reduction of NAD^+^ to NADH 17.

### siRNA

Mature osteoclasts were transfected (RNAiMAX, Invitrogen) with 50 nm siRNA targeting HIF-1*α*, HIF-2*α* or an HIF-1*α* scrambled control. Duplexes were removed after 16 h and osteoclasts incubated for a further 8 h prior to hypoxic stimulation, achieving 75 ± 4% (HIF-1*α*) and 49 ± 6% (HIF-2*α*) protein inhibition, as described 17.

### HRE–luciferase assay

Osteoclasts were transfected with PGK HRE–firefly luciferase (a gift from Professor AL Harris, Oxford, UK) and pHRG–TK *Renilla* luciferase plasmids (Promega), using Lipofectamine 2000 (Invitrogen). 16 h post-transfection, the cells were exposed to experimental conditions. Luminesence was assayed using the Dual-Luciferase Reporter Assay System (Promega), with firefly luciferase normalized to the Renilla transfection control.

### Statistics

Results are expressed as mean ± standard deviation (SD) of at least three independent experiments. Statistical analysis comprised one-way analysis of variance (ANOVA) using Bonferroni's multiple comparison as a *post hoc* test (except for experiments with only two conditions, for which a *t*-test was applied), with results considered significant at *p* < 0.05.

## Results

### Hypoxia increases mitochondrial metabolic activity in osteoclasts

**Figure 1:**
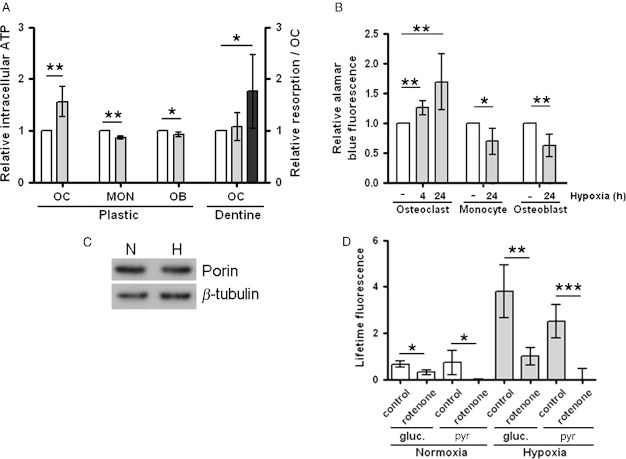
Hypoxia enhances mitochondrial metabolic activity. (A) Intracellular ATP assayed in primary human osteoclasts (OC), monocytes (MON) and osteoblasts (OB) following 24 h of culture in either normoxia (white bars) or hypoxia (2% O_2_, grey bars); (right axis) relative amount of lacunar resorption following 24 h in hypoxia (black bar). Results are normalized to cell number and expressed relative to the normoxic level of ATP/resorption. (B) Relative Alamar Blue fluorescence following culture in normoxia (24 h, white bars) or hypoxia (2% O_2_, 4 h or 24 h, grey bars). Results are normalized to cell number and expressed relative to the normoxic fluoresence. (C) Western blot of osteoclasts following 24 h of exposure to normoxia (N) or hypoxia (2% O_2_, H). (D) Lifetime fluorescence values assessed as an indication of O_2_ consumption rate in normoxic (white bars) and hypoxic (2% O_2_, grey bars) conditions. Osteoclasts were cultured in either medium containing glucose (gluc) or medium lacking glucose but supplemented with 1 mm pyruvate (pyr), with or without rotenone (10 μM). *p < 0.05; **p < 0.01; ***p < 0.001.

To investigate whether hypoxic osteoclasts produce additional energy for bone resorption, we measured intracellular ATP under normoxia and hypoxia (24 h, 2% O_2_). When cultured on plastic, primary monocytes and osteoblasts, which share the osteoclast bone micro-environment, showed reduced intracellular ATP in line with published reports 6, whereas hypoxic osteoclasts increased intracellular ATP by 56% (Figure 1A). When cultured instead on dentine, a substrate on which osteoclast resorption mechanisms are active, the hypoxic increase in intracellular ATP was not evident, suggesting that this ATP is utilized for bone resorption (Figure 1A).

We next assessed mitochondrial metabolic flux, assaying ETC activity using Alamar Blue 25. Hypoxic osteoclasts rapidly increased ETC activity (125%, 4 h), reaching 169% at 24 h compared with decreased ETC activity in monocytes and osteoblasts (Figure 1B). Unaltered mitochondrial porin expression (Figure 1C) and nonyl-acridine orange staining (which binds mitochondrial cardiolipin; data not shown), suggested this was not due to increased mitochondrial mass. O_2_ consumption remained considerable under hypoxia; indeed, ETC inhibition with rotenone had a greater effect under hypoxia (74% reduction) than in normoxic conditions (44% reduction; Figure 1D). In both environments O_2_ consumption via the ETC. remained close to maximal, as compared with cells cultured in supplementary pyruvate (Figure 1D). However, as probe sensitivity to changes in O_2_ concentration is greater in the low O_2_ range, we were unable to compare O_2_ consumption rates at 20% and 2% O_2_ directly.

**Figure 2:**
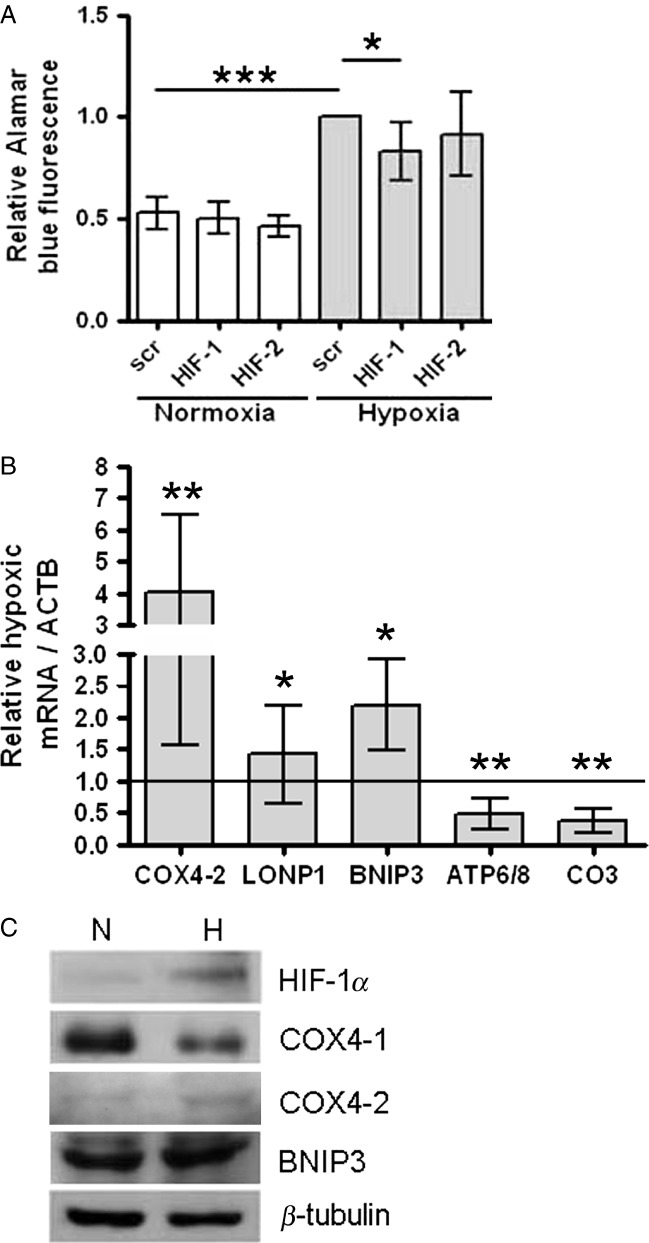
Effect of HIF on mitochondrial metabolic activity. (A) Relative Alamar Blue fluorescence following culture in normoxia (24 h, white bars) or hypoxia (2% O_2_, 24 h, grey bars), following treatment with siRNA targeting HIF-1*α* (HIF-1), HIF-2*α* (HIF-2) or scrambled siRNA control (scr). Results are normalized to cell number and expressed relative to the hypoxic control fluoresence. (B) Hypoxic expression of mRNA versus *β-actin* mRNA (*ACTB*) control, shown relative to the normoxic level of expression. (C) Western blot of osteoclasts following 24 h of exposure to normoxia (N) or hypoxia (2% O_2_, H). *p < 0.05; **p < 0.01; ***p < 0.001.

HIF-1*α* siRNA reduced the hypoxic increase in ETC activity by 25% (Figure 2A), suggesting it to be partially HIF-1*α*-mediated. We therefore considered mitochondrial components of the HIF-mediated metabolic switch. COX subunit switching was evident, with increased COX4-2 (mRNA, protein) and reduced COX4-1 protein due to induction of the mitochondrial protease LONP1 (Figure 2B, C). However, despite hypoxic induction of *BNIP3* mRNA, BNIP3 protein was unchanged (Figure 2B, C). This implies no stimulation of mitophagy, despite reduced expression of mitochondrial mRNAs, ATP synthase F0 subunit a/8 (*ATP6/8*) and cytochrome *c* oxidase subunit 3 (*CO3*) (Figure 2B).

### Hypoxia increases glycolytic flux in osteoclasts

**Figure 3:**
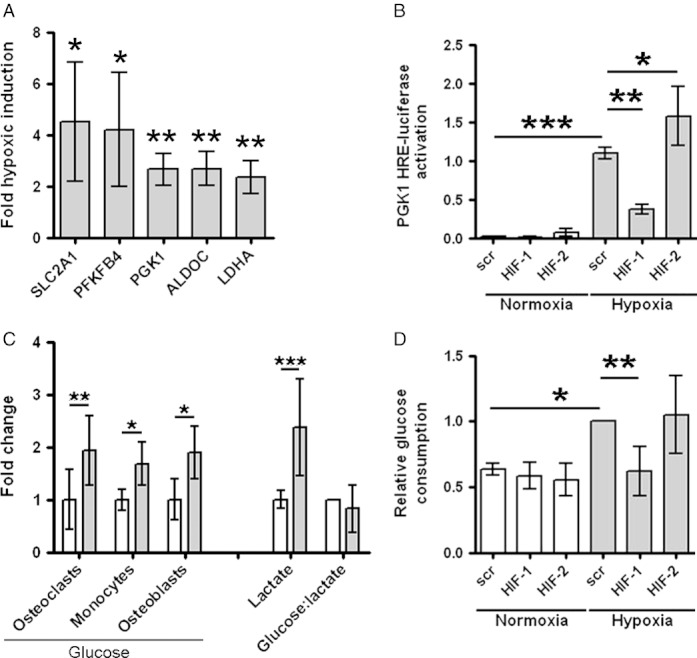
Glucose uptake and glycolysis. (A) Hypoxic expression of mRNA versus *β-actin* mRNA (*ACTB*) control, shown relative to the normoxic level of expression. (B) PGK-1 HRE luciferase activation following 24 h of culture in normoxia (white bars) or hypoxia (2% O_2_, 24 h, grey bars) following treatment with siRNA targeting HIF-1*α* (HIF-1), HIF-2*α* (HIF-2) or scrambled siRNA control (scr). Results are normalized to the *Renilla* transfection control and expressed relative to the hypoxic scrambled control. (C) Glucose consumption, lactate secretion and glucose consumption: lactate secretion ratio following 24 h of exposure to either normoxia (white bars) or hypoxia (2% O_2_, grey bars). Results are normalized to cell number and expressed relative to the normoxic control. (D) Glucose consumption by osteoclasts following 24 h of culture in normoxia (white bars) or hypoxia (2% O_2_, 24 h, grey bars) following treatment with siRNA targeting HIF-1*α* (HIF-1), HIF-2*α* (HIF-2) or scrambled siRNA control (scr). Results are normalized to cell number and expressed relative to the hypoxic control. *p < 0.05; **p < 0.01; ***p < 0.001.

Glycolysis is the major pathway driving osteoclast metabolism. Hypoxia increased glucose transporter (*Glut-1*, *SLC2A1*) and glycolytic enzyme mRNA expression (Figure 3A). Hypoxic transactivation of the PGK-1 hypoxia response element was HIF-1*α*-dependent, while HIF-2*α* exerted a small inhibitory effect (Figure 3B). Hypoxia increased glucose uptake by osteoclasts (and primary monocytes and osteoblasts) in a HIF-1*α*-dependent manner (Figure 3C, D). Hypoxia increased osteoclast lactate secretion, although the ratio of glucose consumption to lactate production was unaltered (Figure 3C), indicative of increased flux through the glycolytic pathway but not of a switch to anaerobic glycolysis.

### Active PDH in hypoxic osteoclasts

**Figure 4:**
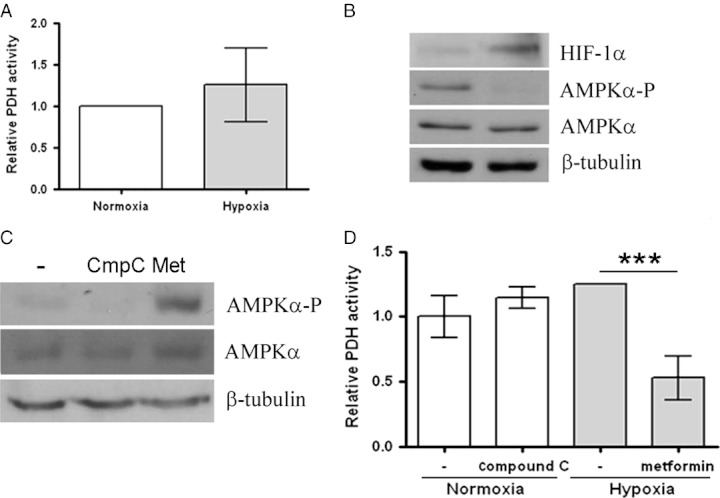
PDH activity. (A) PDH activity following 24 h of exposure to either normoxia (white bar) or hypoxia (2% O_2_, grey bar). Results are normalized to protein concentration and expressed relative to normoxic activity. (B) Western blot of osteoclasts following 24 h of exposure to normoxia (N) or hypoxia (2% O_2_, H); *n =* 2. (C) Western blot following 24 h of exposure of osteoclasts to either compound C (10 μM, CmpC) or metformin (1 mm, Met). (D) PDH activity following 24 h of exposure to either normoxia (white bars) or hypoxia (2% O_2_, grey bars) and either compound C (10 μM) or metformin (1 mm). Results are normalized to protein concentration and expressed relative to hypoxic activity. *p < 0.05; **p < 0.01; ***p < 0.001.

The PDH complex converts pyruvate to acetyl CoA and is permissive for continued mitochondrial metabolic flux. Phosphorylation by the HIF-1*α* target gene PDK1 inhibits PDH activity. Despite hypoxic induction of *PDK1* mRNA (two-fold; *p* < 0.05), no consistent effect of hypoxia was observed on PDK1 protein expression in osteoclasts (data not shown). Hypoxia did not alter PDH activity (Figure 4A) and neither did isoform-specific HIF siRNA affect PDH activity in either condition (data not shown).

We therefore considered whether another regulator of cellular energy status, AMP-activated protein kinase (AMPK), might regulate PDH activity in osteoclasts. AMPK is activated by phosphorylation of Thr172 under metabolically stressful conditions, such as hypoxia 26. However, AMPK phosphorylation was almost ablated in hypoxic osteoclasts (Figure 4B). Chemical inhibition of AMPK with compound C similarly did not affect PDH activity (Figure 4C, D). However, chemical activation of AMPK with metformin inhibited PDH, suggesting that osteoclast AMPK could inhibit PDH activity under hypoxia if it were activated.

### Hypoxia enhances glutamine uptake in osteoclasts

**Figure 5:**
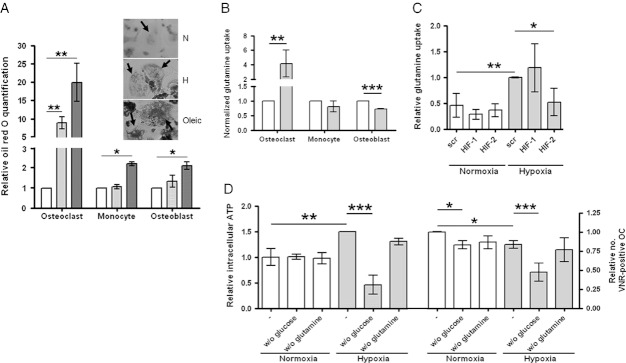
Alternative substrates. (A) Quantified Oil Red O staining following 24 h of exposure to normoxia (N, white bars), hypoxia (2% O_2_, H, light grey) or 100 μM oleic acid (dark grey bars). (Inset) Oil Red O staining of a representative osteoclast preparation (arrows indicate large, multinucleated osteoclasts). (B) Glutamine uptake following 24 h of exposure to either normoxia (white bars) or hypoxia (2% O_2_, grey bars). Results are normalized to cell number and expressed relative to normoxic uptake. (C) Glutamine uptake following 24 h of culture in normoxia (white bars) or hypoxia (2% O_2_, 24 h, grey bars) following treatment with siRNA targeting HIF-1*α* (HIF-1), HIF-2*α* (HIF-2) or scrambled siRNA control (scr). Results are normalized to cell number and expressed relative to the hypoxic control. (D) Intracellular ATP and osteoclast number assayed following 24 h of culture in either normoxia (white bars) or hypoxia (2% O_2_, grey bars), with or without (w/o) either glucose or glutamine. ATP results are normalized to cell number and expressed relative to the hypoxic level of ATP. *p < 0.05; **p < 0.01; ***p < 0.001.

We next considered other substrates of mitochondrial metabolism that might contribute to hypoxic ATP generation. Fatty acid oxidation has been proposed to drive ATP production in osteoclasts 23. However, hypoxic osteoclasts accumulated intracellular neutral lipid (Figure 5A), suggesting that hypoxia inhibits mitochondrial import and/or utilization of fatty acids 27,28. Hypoxia did not affect lipid accumulation in monocytes or osteoblasts.

Glutamine, a substrate for glutaminolysis, enters the mitochondrial TCA cycle at *α*-ketoglutarate. Hypoxic osteoclasts increased glutamine uptake 4.1-fold, whereas osteoblasts and monocytes inhibited and maintained uptake respectively (Figure 5B). Hypoxic induction of glutamate uptake was HIF-2*α*-dependent (Figure 5C). However, glutamine withdrawal had no effect on either ATP production or osteoclast survival, whereas removal of glucose dramatically inhibited both, especially in hypoxia (Figure 5D).

### Osteoclasts are sensitive to hypoxia-induced cell death

**Figure 6:**
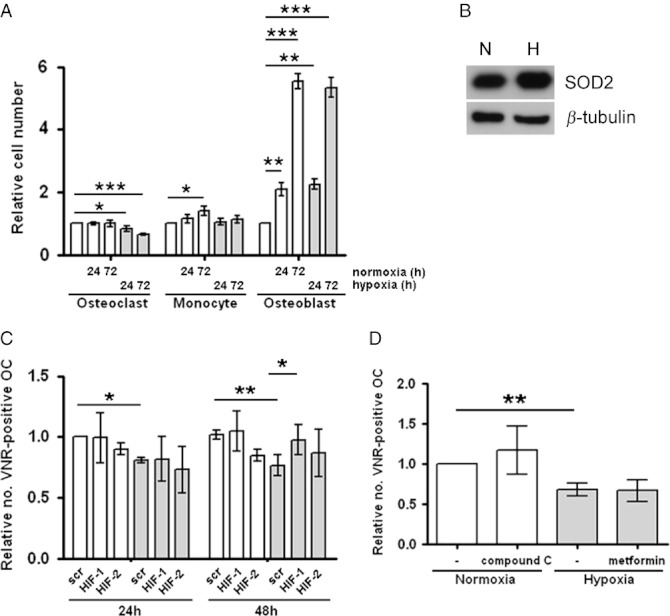
Osteoclast survival. (A) Relative number of osteoclasts, monocytes and osteoblasts following 24 h or 72 h of exposure to either normoxia (white bars) or hypoxia (2% O_2_, grey bars). (B) Western blot of osteoclasts following 24 h of exposure to normoxia (N) or hypoxia (2% O_2_, H). (C) Relative number of osteoclasts following 24 h or 48 h of exposure to either normoxia (white bars) or hypoxia (grey bars) following treatment with siRNA targeting HIF-1*α* (HIF-1), HIF-2*α* (HIF-2) or scrambled siRNA control (scr). (D) Treatment with compound C (10 μM) or metformin (1 mm). *p < 0.05; **p < 0.01; ***p < 0.001.

In contrast to other cells, osteoclasts are sensitive to even moderate hypoxia. 2% O_2_ reduced osteoclast numbers to 83% and 65% of normoxic levels at 24 and 72 h respectively, whereas primary monocytes and osteoblasts continued to proliferate (Figure 6A). We previously hypothesized the extent of osteoclast-mediated bone resorption under hypoxia to be a balance between osteoclast activation and osteoclast apoptosis 14. Sensitivity to hypoxia-induced cell death might be a consequence of maintaining high rates of oxidative phosphorylation in a hypoxic environment, evidenced by increased expression of superoxide dismutase 2 (SOD2) (Figure 6B), a marker of mitochondrial ROS formation. HIF-1*α* siRNA completely rescued osteoclasts from cell death induced by chronic (48 h) hypoxic exposure (Figure 6C), whereas modulation of AMPK activity had no effect (Figure 6D).

## Discussion

We have demonstrated that hypoxic osteoclasts increase flux through both the glycolytic pathway and the mitochondrial ETC, driven by employment of selected components of the HIF-1*α*-mediated switch to anaerobic glycolysis. This enables osteoclasts to generate sufficient ATP to support enhanced bone resorption.

Glucose regulates osteoclast activity at normal physiological concentrations 21. Increased glucose rapidly elevates the intracellular ATP:ADP ratio 22, while longer exposure activates vacuolar H^+^-ATPase (V-ATPase) A-subunit transcription to stimulate resorption 29. This subunit directly interacts with the glycolytic enzyme phosphofructokinase-1 (PFK-1) 30. Interaction between aldolase and the V-ATPase E-subunit also occurs in osteoclasts 31. Both interactions are deemed mechanisms to micro-compartmentalize glycolytic ATP generation at the required intracellular location, directly linking glycolysis and osteoclast activation. Increased glycolysis by actively resorbing, hypoxic osteoclasts may also occur *in vivo*. Positron emission tomography (PET) with 2-(fluorine-18)fluoro-2-deoxy-d-glucose (^18^FDG) can distinguish benign primary bone tumours containing many osteoclasts from those where osteoclasts are sparse 32, additionally correlating with markers of hypoxia 33. Agents which inhibit glucose uptake/glycolysis inhibit bone resorption in animal models of disease 34,35 and can induce clinical remission in RA 36, suggesting targeted inhibition of glycolysis as a therapeutic anti-resorptive option. However, other cells within the hypoxic rheumatoid joint exhibit increased glycolytic capacity and the inhibitory activity of these agents does not solely target glycolysis, necessitating further investigation of the mechanism(s) behind such results.

Glutamine consumption has not previously been described in osteoclasts. Hypoxia increased glutamine uptake, as in SK–N–SH neuroblastoma 37 and A549 lung adenocarcinoma cells 38, in a HIF-2*α*-dependent manner. HIF-2*α* also drives the switch to hypoxic use of glutamine, rather than glucose, as the substrate for lipid synthesis 38. Glutamine is additionally required for nucleotide and hexosamine biosynthesis. This complements our data that hypoxic osteoclasts require glucose for ATP production and cell survival but are insensitive to glutamine withdrawal, suggesting glucose as the primary substrate for ATP production, with glutamine potentially used for biosynthesis.

Osteoclasts only utilized selected components of the classical HIF-1*α*-mediated metabolic switch to anaerobic respiration. HIF-1*α* increased hypoxic glucose uptake and glycolytic flux and the HIF-1*α*-dependent COX subunit switch was observed. However, PDH was not inhibited and BNIP3 expression did not increase. These latter processes normally reduce hypoxic flux through the mitochondrial TCA cycle and ETC 10,11 and induce mitochondrial autophagy 12 respectively, both of which prevent toxic accumulation of ROS. As a result, mitochondrial ROS accumulates in hypoxic osteoclasts. However, ROS are essential for resorption. Tartrate-resistant acid phosphatase (TRAP) and cathepsin K co-localize in transcytotic vesicles. Digestion of TRAP by cathepsin K activates the ROS-generating activity of TRAP to aid degradation of the products of bone digestion 39. Osteoclast differentiation and resorption are enhanced by exogenous inducers of ROS; including H_2_O_2_ 40, homocysteine 41 and hypoxia 42. Indeed, this effect of hypoxia requires mitochondrial ROS and is reversed by the mitochondria-specific antioxidant MitoQ 42.

It is unclear why PDH was not inhibited in hypoxic osteoclasts. Despite HIF-1*α* induction, *PDK1* mRNA was only modestly induced and we observed no consistent effect on PDK1 protein. PDH is also inhibited by hypoxic activation of AMPK and induction of PDK4 43. However, in osteoclasts hypoxia inhibited AMPK phosphorylation and so inactivated AMPK. AMPK is usually activated by reduced intracellular ratios of ATP:ADP or ATP:AMP, although hypoxic activation occurs independently of changes in intracellular energy status 26,44. It may be that enhanced ATP production in hypoxic osteoclasts increases the intracellular ratio of ATP:AMP and overrides hypoxic mechanism(s) of AMPK activation in favour of dephosphorylation and inactivation. This would be necessary for hypoxic resorption to proceed, as AMPK inhibits osteoclast differentiation and activation 45. Hypoxic attenuation of PDH activity is therefore prevented in osteoclasts by blockade of at least two pathways that usually contribute to its inhibition, allowing continued mitochondrial metabolic flux under hypoxia.

HIF-1*α* did not reduce mitochondrial metabolic flux, but did increase glycolytic flux and maintain the COX subunit switch, supporting our observation that increased hypoxic mitochondrial activity is partially HIF-1*α*-dependent. HIF-1*α* siRNA also prevented cell death during prolonged hypoxia. We hypothesize that in hypoxic osteoclasts, functional HIF-1*α*-dependent pathways initially increase ATP production and bone resorption 14. However, lack of activation of HIF-1*α*-dependent survival pathways eventually results in cell death. As osteoclasts are anyway short-lived cells, and as resorption cannot be allowed to continue indefinitely, allowing progressive accumulation of ROS in hypoxic osteoclasts may be an adaptive mechanism permitting rapid bone resorption in the short term, while ensuring curtailment of the process in the absence of re-oxygenation.

In summary, we have shown that hypoxia stimulates osteoclasts' consumption of glucose and glutamine and that increased glucose uptake is required for increased ATP production. HIF-1*α*-dependent stimulation of glucose uptake and glycolysis, in the absence of PDH inhibition by either HIF-1*α* or AMPK, drives high hypoxic mitochondrial ETC activity in these cells, although this eventually results in cell death. These mechanisms appear to support hypoxic induction of osteoclast resorption in the short term and might provide therapeutic targets for amelioration of the pathological bone resorption associated with diseases such as rheumatoid arthritis.

## SUPPLEMENTARY MATERIAL ON THE INTERNET

The following supplementary material may be found in the online version of this article:

Additional methodology for O_2_ consumption assay
