# Supplementary material for: Differential regulation of HIF-mediated pathways increases mitochondrial metabolism and ATP production in hypoxic osteoclasts
Source: J Pathol. 2013 Mar 13;229(5):755–64. doi: 10.1002/path.4159 (PMC3618370; doi:10.1002/path.4159)
Supplement: Supplementary file 1 [file path0229-0755-SD1.doc]

+A: **Additional methodology for oxygen consumption assay**

Assays were performed on osteoclasts in black 96-well plates with clear bottoms. Lifetime measurements of oxygen consumption were carried out to reduce run variability, using a bottom-read function with the TR EX and TR EM filters set for excitation and emission to give maximum sensitivity. Integration start times of 30 s and 70 µs were used, with a measurement integration time of 30 µs. Data was processed using the script mode function on the BMG FLUOstar Omega reader.
